# MicroRNAs and lncRNAs—A New Layer of Myeloid-Derived Suppressor Cells Regulation

**DOI:** 10.3389/fimmu.2020.572323

**Published:** 2020-10-02

**Authors:** Elham Safarzadeh, Zahra Asadzadeh, Sahar Safaei, Arash Hatefi, Afshin Derakhshani, Francesco Giovannelli, Oronzo Brunetti, Nicola Silvestris, Behzad Baradaran

**Affiliations:** ^1^Department of Microbiology & Immunology, Faculty of Medicine, Ardabil University of Medical Sciences, Ardabil, Iran; ^2^Immunology Research Center, Tabriz University of Medical Sciences, Tabriz, Iran; ^3^Department of Pharmaceutics, Rutgers, The State University of New Jersey, Piscataway, NJ, United States; ^4^Medical Oncology Unit—Istituto di Ricovero e Cura a Carattere Scientifico (IRCCS) Istituto Tumori “Giovanni Paolo II” of Bari, Bari, Italy; ^5^Department of Biomedical Sciences and Human Oncology, Department of Internal Medicine and Oncology (DIMO)—University of Bari, Bari, Italy; ^6^Department of Immunology, Faculty of Medicine, Tabriz University of Medical Sciences, Tabriz, Iran

**Keywords:** myeloid-derived suppressor cells, cancer, miRNA, tumor microenvironment, long non-coding RNAs, molecular mechanism

## Abstract

Myeloid-derived suppressor cells (MDSCs) constitute an important component in regulating immune responses in several abnormal physiological conditions such as cancer. Recently, novel regulatory tumor MDSC biology modulating mechanisms, including differentiation, expansion and function, were defined. There is growing evidence that miRNAs and long non-coding RNAs (lncRNA) are involved in modulating transcriptional factors to become complex regulatory networks that regulate the MDSCs in the tumor microenvironment. It is possible that aberrant expression of miRNAs and lncRNA contributes to MDSC biological characteristics under pathophysiological conditions. This review provides an overview on miRNAs and lncRNAs epiregulation of MDSCs development and immunosuppressive functions in cancer.

## Introduction

Myeloid-derived suppressor cells (MDSCs) are produced due to aberrant myelopoiesis (1) and represent one of the most pivotal mediators in the orchestration of immunosuppression (2). The normal development and maturation of myeloid linage cells are essential for the promotion of innate immunity, however, under pathological contexts such as chronic inflammation or cancer, normal hematopoiesis is dysregulated, signals that origin from the HSCs niche alter the magnitude and combination of the hematopoietic output, a characteristic of immune regulation known as “emergency” hematopoiesis, needed to provide appropriate supply of both myeloid and lymphoid cells to increased demand (3). In particular, in cancer, modified myelopoiesis generates lineage-restricted hematopoietic progenitors, resulting in the accumulation of myeloid cells with an immature state and more importantly with the characteristic as an immune response suppressive in the bone marrow (4, 5).

Of note, MDSC are considered as not only immature myeloid cells but also matured cells that could be converted to MDSC. Rivoltini et al. reported that culturing monocytes with melanoma-derived exosomes *in vitro* generates cells which have phenotypic and functional characteristics similar to those exhibited by MDSC (Exo-MDSC). It was indicated that Exo-MDSC have an increased level of mRNA and release of protumourigenic and immunosuppressive cyto/chemokines, decrease the expression of HLA-DR, and suppress T cell proliferation. This hypothesis is well-supported by the evidence that peripheral MDSC from melanoma patients share characteristics of gene-expression and miRNA profile, phenotypic and functional features of Exo-MDSC (6).

MDSCs are accumulated and expand in the tumor microenvironment and in the peripheral lymphoid organs of tumor-bearing hosts (7–9). Several studies indicated the mechanisms of MDSC-mediated immune suppression and its expansion (10, 11). Ongoing investigation has shown epigenetic regulation of MDSCs as a potential approach in attaining this objective. Indeed, epigenetics states all heritable phenotype variations in gene expression (active vs. inactive genes) without any modifications in the underlying DNA sequence. The mentioned epigenetic modifications enable substantial adjustability in the gene expression, rather than just genes to be turned off or on. DNA variation, covalent histone change, and RNA interference are three systems, fundamental in starting and maintaining epigenetic silencing. Epigenetic reprogramming of MDSC's properties results in the alteration of its features, with a consequential rearrangement of the tumor milieu instead of opposing the development and progression of the tumor (12).

More recently, several studies have also unveiled an important regulatory role for miRNAs and long non-coding RNAs (lncRNA) in modulating immune responses and in MDSCs differentiation and functions (13–17). This review will summarize the miRNAs and lncRNAs that contribute to the differentiation, maturation, and immunosuppressive function of MDSCs in cancer. With this goal in mind, this review will summarize miRNAs and lncRNAs that contribute to MDSCs differentiation, maturation, and immunosuppressive function in cancer.

## Myeloid-Derived Suppressor Cells

Major studies introduce MDSCs as a heterogeneous population of cells with myeloid origin (18). The heterogeneity features of such cells including their phenotype and functions are highly influenced by the type of tumor and the stage of tumor progression (19). MDSCs are described in animals and humans with malignancy (20, 21). MDSCs in mice are characterized by co-expression of the CD11b (CR3A or integrin αM) and GR1,myeloid-cell lineage differentiation antigen, comprising Ly6C and Ly6G isoforms and divided in two subpopulations polymorphonuclear (PMN)-MDSC (CD11b^+^Ly6G^+^Ly6C^lo^) and monocytic (M)-MDSC (CD11b^+^Ly6G^−^Ly6C^hi^) (22– 24).

According to a new classification by Bronte et al., human MDSCs are generally classified as follows: PMN-MDSC described as CD11b^+^CD14^−^CD15^+^ or CD11b^+^CD14^−^CD66b^+^ and M-MDSC described as HLA-DR^−/lo^CD11b^+^CD14^+^ CD15^−^. Another population also described as early-stage MDSC (eMDSC) and characterized by the HLA-DR^−^ CD33^+^ Lin^−^ (CD3, CD19, CD56, CD14, and CD15) phenotype, includes mixed cells of MDSC comprising more immature progenitors (25–27). Moreover, Fibrocytic MDSCs (F-MDSCs), a novel MDSC subset, were recently described as CD11b ^low^ CD11c ^low^ CD33^+^ Interleukin (IL)-4Ra^+^ cells with fibrocystic phenotypes (28, 29). In tumor-bearing mice and cancer patients, although PMN-MDSCs are the dominant subsets (70–80%) of the total MDSCs population, this subset demonstrates less immunosuppressive features than M-MDSCs (5, 30).

Several studies suggested that MDSCs represent a “dysfunctional state” of myeloid lineages such as monocytes/neutrophils (31). Dysregulation in myelopoiesis in cancerous and non-cancerous conditions induces MDSC differentiation and expansion and affects the host immune response (22, 32). In the case of cancer, the tumor cells produce different mediators that inhibit the differentiation of mature myeloid cells and, in parallel, stimulate MDSCs expansion, generating an immunosuppressive microenvironment that affects tumor progression (33). These mediators include bombina variegata peptide 8 (Bv8), KIT ligand [stem-cell factor (SCF)], prostaglandin E2 (PGE2), vascular endothelial growth factor (VEGF), FMS-like tyrosine kinase 3 ligand(FLT3L), granulocyte colony-stimulating factor (G-SCF), macrophage colony-stimulating factor(M-CSF), granulocyte-macrophage colony stimulating factor (GM-CSF), IL-1, IL-6, TNF, and IL-10. It was shown that GM-CSF and IL-6 could be the most powerful expanding factors of MDSCs deriving from bone marrow progenitors (11, 34–37). In addition, the immunosuppressive activity of MDSCs requires mediators which induce their activation (38). The most important MDSC activating mediators are the Transforming Growth Factor (TGF-ß), ligands for toll-like receptors, IL-1β, Interferon (IFN)-γ, and IL-4 which are chiefly originated from activated T cells and tumor stromal cells. Most of these mediators promote signaling pathways that converge the Signal transducer and the activator of transcription (STAT)-1, STAT-6, and nuclear factor-κB (NFκB) signaling pathways (4, 39–43).

MDSCs are involved in determining potent immune-negative regulation and tumor-promoting functions in the TME via multiple mechanisms. MDSCs could deplete and capture the amino acids required for T cell activation and proliferation such as L-arginine and L-cysteine, thus generating the reactive oxygen and nitrogen species such as NO, ROS, and peroxynitrite (44–46). Activated M-MDSCs upregulate Arginase-1 (Arg1) and inducible nitric oxide synthase (iNOS), while activated PMN-MDSCs highly express Arg1 and reactive oxygen species (ROS) (47, 48). High expression of Arg1 causes the depletion of L-arginine resources, resulting in T-cell function impairment and inhibition of T cell proliferation via several mechanisms, including translational blockade of CD247 [the ζ chain of the T-cell receptor (TCR)] and decreased production of IFN-γ/IL-2 by T cells (30, 49–52). Arginine is also oxidized by iNOS to produce citrulline and Nitric Oxide (NO) (53). NO, a powerful oxidative modulator, can inhibit T cell proliferation, adhesion, and migration by blocking Jak3 and STAT5 transcription factor activity, inhibiting E-selectin expression on endothelial cells and inducing T-cell apoptosis (54, 55). NO is also changed to the radical peroxynitrite, a reactive nitrogen-oxide species, in the presence of ROS (56). Peroxynitrite alters tyrosine side chains in the TCR-CD8 complex, prevents peptide-MHC-TCR interaction and finally stops T cell responses (57). ROS play a fundamental role in the induction of T cell apoptosis through B cell lymphoma-2 (Bcl-2) downregulation (58). Moreover, by producing indoleamine 2,3 dioxygenase (IDO) and reducing local tryptophan levels, MDSCs can inhibit T cells proliferation (59).

MDSCs also exert immunosuppressive features by skewing anti-tumor immune cells subsets toward immune suppressive counterparts, for example, through the conversion of naïve CD4^+^ T cells into Tregs and polarization of macrophages toward an alternatively activated macrophages (M2) phenotype (22, 60). As a matter of fact, MDSCs through the production of IL-10 and TGF-β, arginine deprivation and CD40-CD40L interacting can induce Treg cells (61). Chen et al. (62) have demonstrated that TGF-β production leads to Foxp3 gene expression in T cells, which in turn re-polarizes them toward a Treg phenotype with a potent immunosuppressive potential. Moreover, activation of M2 macrophages due to factors like IL-13, IL-10, IL-4, and glucocorticoid, promote tumor growth by secreting high levels of IL-10 and low levels of IL-12. MDSC-polarizes TAMs toward the M2 phenotype via the production of IL-10 in which it suppresses IL-12 and enhances IL-10 production by these cells. These polarized cells create a feedback cycle through MDSCs modification and enhance IL-10 production, which will subsequently facilitate the tumor growth (63).

MDSCs can also down-regulate homing receptors on the surface of T cells, such as L-selectin (CD62L), by expression of aADAM17 (disintegrin and metalloproteinase domain 17) as well as inhibit T cell homing to lymph nodes (52).

## Micro RNAs

**Figure 1:**
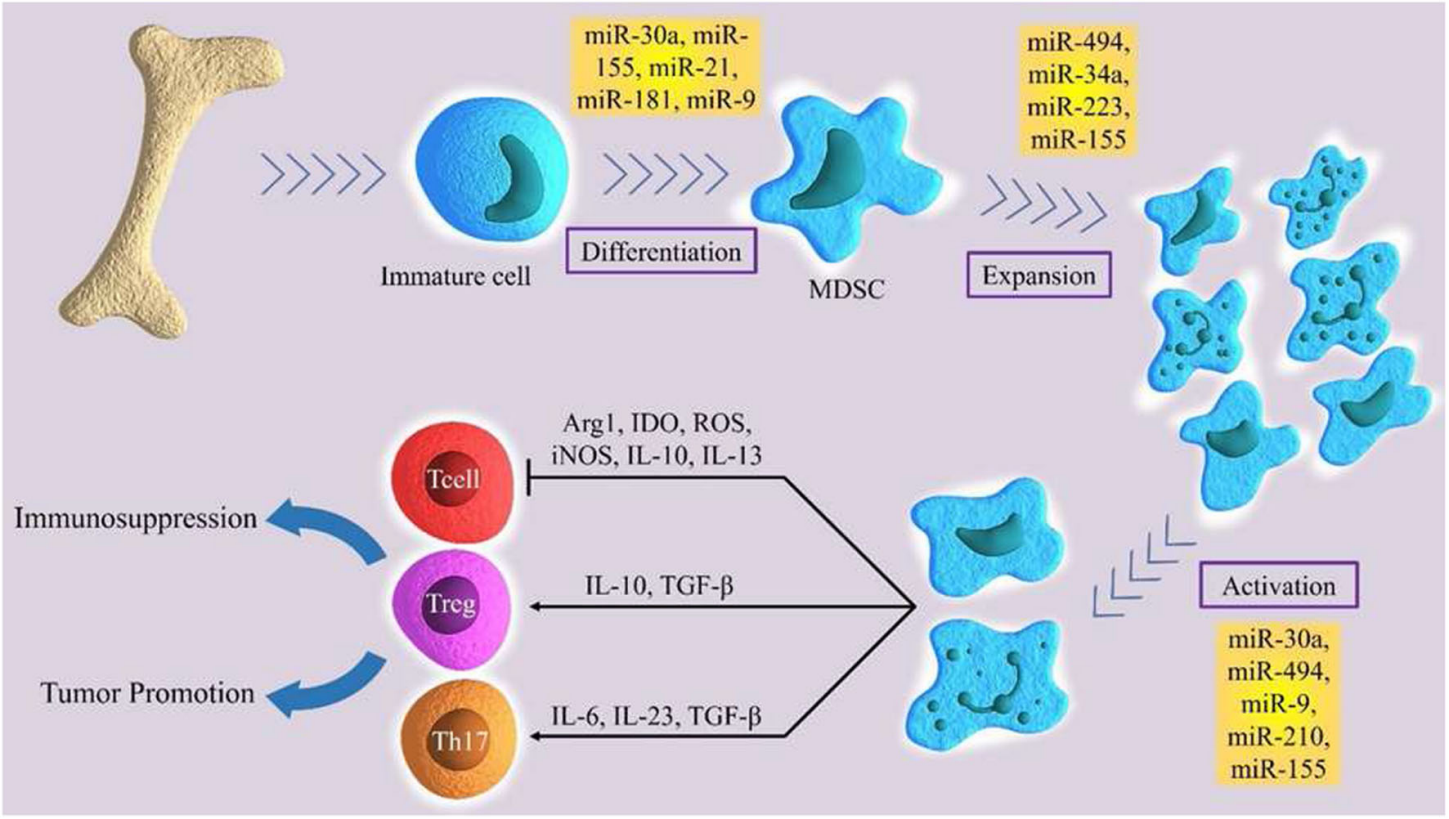
The role of certain miRNAs in regulation of differentiation, expansion and activation of MDSCs.

MicroRNAs are small, single-stranded, non-coding RNAs of approximately 22 nucleotides (64). miRNAs control about 30% of human genes (65). They regulate range of physiological processes, such as cell cycle, differentiation, development, and metabolism (66–69), as well as pathological process, such as chronic disease, immuno- or neurodegenerative diseases, and cancer (70–74). MiRNAs are progressively being described as main factors in regulating the immune system. They exert an important role in the epiregulation of the development, the differentiation and the activity of various immune cells, like B and T lymphocytes, dendritic cells, and macrophages (75–78). Moreover, miRNAs show a notable regulatory role in the development, expansion, and function of MDSC (Figure 1) (79). Several studies demonstrated the main role of several miRNA in cancer development. In particular, these molecules have been studied in order to identify potential diagnostic, prognostic or therapeutic targets in several tumors (80, 81). Numerous current researches emphasize the function of certain miRNAs in the MDSCs regulation (Table 1).

**Table 1:** Summary of miRNAs implicated in regulation of MDSC development and function.

**miRNA**	**Target gene(s)**	**Disease**	**Function**	**References**
miRNA-30a	SOCS3	B-cell lymphoma	Increases MDSC differentiation and immunosuppressive activity	(82)
miRNA-494	PTEN	Murine breast cancer	Activates the PI3K/Akt pathway and increases activity and accumulation of MDSCs	(83, 84)
miRNA-200c	PTEN and FOG2	–	Activates STAT3 and PI3K/Akt and augments immune suppressive activity of MDSCs	(85)
miRNA-9	Runx1	Lewis lung carcinoma	Reduces MDSC differentiation and increases MDSC-mediated suppression	(86)
miRNA-210	IL-16 and CXCL12	–	Enhances MDSC activity through augmenting arginase function and generation of nitric oxide	(87)
miRNA-34a	N-myc	–	Inhibits MDSCs apoptosis by suppressing the expression of N-myc	(88)
miRNA-223	Mef2c Arg1 and Stat3	Breast cancer cell murine model of autoimmune encephalomyelitis	Decreases accumulation of MDSCs by inhibits progenitor expansion Enhances accumulation of MDSC and lower levels of CNS-isolated T cell responses	(89–91)
miRNA-155	SHIP-1 &SOCS-1	–	Development of MDSC and also for MDSC-mediated modulation of CD4^+^Foxp3^+^ regulatory T cells to facilitate tumor growth	(92, 93)
miRNA-21 and miRNA-181	NFI-A	–	Promotes MDSC generation and their inhibition reduces MDSCs and provokes myeloid differentiation in macrophage and dendritic cells	(94, 95)

*MDSC, myeloid-derived suppressor cells; SOCS3, Suppressor of cytokine signaling 3; PTEN, Phosphatase and tensin homolog; FOG, Friend of GATA2; MEF2C, Myocyte Enhancer Factor 2C; NFIA, nuclear factor I A*.

### MicroRNA-30a

A recent study revealed that miR-30a as a member of the miR-30 family increases the differentiation of MDSCs and their immunosuppressive activity through regulating SOCS3 in B-cell lymphoma model mice. Xu et al. (82) detected augmented expression of miR-30a in PMN-MDSCs and M-MDSCs in mice with B cell lymphoma. MicroRNA-30a targets SOCS3, which is a JAK2/STAT3 pathway regulator. SOCS3 negatively regulates MDSCs development and functions by inhibiting STAT3 activation (96). Reduced SOCS3 expression and stimulated JAK2/STAT3 signaling increase MDSCs differentiation and promotes its suppressive function. In both M-MDSCs and PMN-MDSCs, miR-30a is able to promote the expression of suppressive factors, such as arginase-1, IL-10, and ROS, by targeting SOCS3 and stimulating JAK2/STAT3 signaling, thus resulting in tumor growth, which correlates with increased levels of MDSC in the tumor microenvironment, and reduced CD8^+^ T-cell infiltration in tumors. Furthermore, miR-30a improved the development of tumor by promoting the differentiation and immunosuppressive ability of MDSCs in mice with B-cell lymphoma. MicroRNA-30a antagomir injection in mice demonstrated a considerably improved B-cell lymphoma, while miR-30a agonist injection in mice with B-cell lymphoma stimulated tumor growth (96). These data showed that targeting miR-30a leads to reduced suppression activity of MDSC and lessens the MDSCs numbers, consequently improving anti-tumor response.

### MicroRNA-494

MiR-494 is overexpressed by tumor-derived factors and it can regulate the immunosuppressive function of MDSCs (83). Liu et al. reported that tumor cells secrete TGF-β1 which regulates MDSCs activity via stimulation of miR-494 expression; this stimulation is Smad 3 pathway-dependent. An increased miR-494 expression was noticed in MDSCs in tumor-bearing mice compared with those in tumor-free mice. This research found that miR-494 targets phosphatase and tensin homolog (PTEN) and stimulates the PI3K/Akt pathway and controls the accumulation and function of MDSCs. The PTEN downregulation and augmented activation of the PI3K/Akt pathway changed the intrinsic apoptotic/survival signal and also heightened the effectiveness of CXCR4-related recruitment and increased MDSC immunosuppressive function. This study implied that the stimulated PI3K/Akt pathway strongly supports the matrix metalloproteinase (MMPs) expression and results in tumor invasion and metastasis. Additionally, knockdown of miR-494 considerably reversed MDSC function and tumor development and repressed metastasis in an *in vivo* mouse model of 4T1 mammary carcinoma (84). The data revealed that miR-494 induction by TGF-b1 regulates MDSC accumulation and activity and can be recognized as a possible target in the treatment of cancer.

### MicroRNA-155

MicroRNA-155 is a typical miRNA involved in numerous processes in molecular biology, regulating inflammation, hematopoiesis, and immunity (97). Bioinformatics analyses of functional aspects conducted by Robert et al. indicated that multiple miRNAs, including miR-155, are highly expressed in early hematopoietic progenitors and control early stages of hematopoietic differentiation (98). Normal mature B cells, T cells, granulocytes, and monocytes express low levels of miR-155 (99). Moreover, several findings have supported a critical function for miR-155 in concert to MDSCs, emerging as a critical element in the control of their differentiation and function (100). This molecule is markedly elevated in both PMN-MDSCs and M-MDSCs stimulated with GM-CSF and IL-6. Moreover, the expression of miR-155 in MDSCs was controlled by the TGF-β signaling pathway (100). The induction of the miR-155 expression by TGF-β in turn results in the inhibition of Src Homology Inositol Phosphatase (SHIP-1) as a target of miR-155. SHIP-1 is an adaptor protein that negatively controls the survival and proliferation of myeloid cells. During the induction of functional MDSC, down-regulation of this essential molecule promotes STAT3 activation and accordingly induces MDSC expansion (100). Similarly, an experimental study revealed that genetic ablation of miR-155 confers resistance to the growth of transplanted cancer cell in mice tumor models. Moreover, miR-155 is essential for the development of MDSC and also for MDSC-mediated modulation of CD4^+^Foxp3^+^ regulatory T cells, indicating that MDSCs are associated with miR-155 to promote tumor growth (92). On the other hand, miR-155 deficiency reduces the immune responses of both dendritic cells and T cells against tumor cells. These discrepancies underscore a context-dependent activity of miR-155 in regulating tumor immunity via distinct subsets of immune cells within the tumor microenvironment (92). Although the above data support a role for miR-155 expression in tumor progression, Wang et al. (93) stated that miR-155 deficiency enhances solid tumor development by augmenting the tumor-supporting properties and recruitment of MDSCs to the tumor milieu. These contradictory observations might be associated with the tumor type and the adjacent microenvironment. Future studies are required to clarify the subject.

### MiRNA-34a

Unlike its family members miR-34b/c, miR-34a is expressed in a wide variety of normal human tissues (101). As an example, miR-34a, produced by FoxP3-expressing regulatory T (Treg) cells, has been shown to have a vital role in regulating immune reactions. Yang et al. (102) explored TGF-β signaling activity during hepatocellular carcinoma (HCC) progression, and the results revealed that miR-34a expression is directly regulated by the TGF-β pathway in HCC cell lines. In all HCC cell lines investigated, the stimulation of the TGF-β signaling pathway induced a significant reduction in the expression of miR-34a, leading to the increased production of a miR-34a target gene, the chemokine CCL22, which results in the accumulation of Treg cells which enhance immune escape (102). However, as far as myeloid suppressor cells are concerned, miR-34a resembles a tumor suppressor, since miR-34a-mimic transfected into a murine colorectal carcinoma cell line, CT26 cells, leads to a lower induction of myeloid precursor cells into Gr1^+^CD11b^+^ cells, the phenotype of MDSC with immunosuppressive activity, in mice (103). This finding indicates the negative activity of miR-34a in the induction of MDSC. Consistent with the above data on the correlation of TGF-β and miR-34a expression in Treg cell recruitment, miR-34a might control the action of tumor cells, inducing MDSCs via TGF-β and/or IL-10 (103). Moreover, miR-34a repressed MDSC apoptosis by inhibiting N-myc expression, but did not influence MDSCs proliferation. The over-expression of miR-34a results in an increase of MDSCs due to a reduction of MDSCs apoptosis. The proto-oncogene N-myc which is a transcription factor that has a role in activities, including cell growth, differentiation, and apoptosis, is a target of miR-34a in MDSCs (88). As a result, restoring miR-34a expression contemporarily to stopping TGF-β signaling might offer a promising molecular treatment in concert with MDSC inhibition in cancer.

### MiRNA-21 and miRNA-181

The miR-181 family has a crucial role in normal myeloid differentiation and adult acute myeloid leukemia (AML) development (104). The miR-181 family consists of four miRs: miR-181a/b/c/d. Overexpression of all of them has been detected in different subtypes of adult AML, suggesting an oncogenic role by the direct inhibition of genes involved in myeloid differentiation such as protein kinase C delta (PRKCD) (104). Also, miR-21 is one of the most studied miRNAs that has attracted particular attention in the myeloid subject area. In particular, miR-21 has been identified as a molecule whose deregulation impairs myelopoiesis (94).

Sepsis is a life-threatening condition characterized by activation of the host's inflammatory pathways. Bacterial sepsis is a scenario where pathological inflammation driving defects in innate immunity can result in bacterial growth. However, less reports describe the regulation of miR21 and miR-181 family expression in tumor-induced immunosuppression by MDSCs, the role of these miRNAs in sepsis-related immunosuppression mediated by MDSCs is well characterized. These investigations provide clues from which we may be able to trace their role in development of MDSCs in the course of human malignancies that resemble some disturbances found in sepsis. In sepsis-induced inflammatory response, the produced miR-21 and miR-181b promotes MDSC generation. Inhibition of this miRNAs decreases the population of MDSCs and significantly enhances late-sepsis survival (95). Importantly, concurrent inhibition of both miR-21 and miR-181b in Gr1^+^CD11b^+^ myeloid cells was able to enhance myeloid differentiation into macrophage and dendritic cells and eliminated MDSCs, leading to an enhancement of the innate immunity and improved bacterial clearance. The findings have also linked miR-21 and miR181b with the expression of NFI-A, a transcription factor involved in myeloid differentiation in sepsis (95). These miRNAs upregulate NFI-A, thereby maintaining the myeloid progenitor cells in an immature state (105). This regulatory mechanism underlying the expression of miR-21 and miR-181b in MDSCs under the same pathological condition was further uncovered in a later study. The authors demonstrated that transcriptional regulation of miR-21 and miR-181b is mediated by a combination of factors including STAT3 and C/EBPβ as critical transcription factors in Gr1^+^CD11b^+^ myeloid cells in septic mice. STAT3 and C/EBPβ factors bind to miR-21 and miR-181b and promote and activate the expression of these molecules through a pathway that requires IL-6 signaling, implicating phosphorylation on the Rb protein, ultimately leading to MDSC expansion (106). A more recently published study has provided some more details on the mechanisms of miR-21/miR181 functions on MDSC biology in cancer which involves the suppression of the Mixed-lineage leukemia 1 (MLL1) complex that is found to play an important role in regulating hematopoietic stem cell homeostasis. Mechanistically, tumor-secreted factors and GM-CSF + IL-6 signaling activates Stat3 and Cebpβ leading to the expression of miR-21a, miR-21b, and miR-181b. The induction of these microRNAs inhibits the expression of the components of MLL1 complex and thus may play a critical role in PMN-MDSC expansion, activation, and differentiation (107). In addition, miR-21 expresses some common biological functions with the aforementioned miRNA, miR-155, during MDSC induction in tumor bearing mice (100). In this aspect, the cytokine, TGFβ has been shown to promote the induction of MDSC by upregulating the expression of miR-21 and miR-155. MiR-155 and miR-21 synergistically function to promote MDSC stimulation through targeting SHIP-1 and phosphatase and tensin homolog, respectively, leading to STAT3 activation and MDSC expansion.

### MicroRNA-200c

Also MiR-200c can regulate the suppressive activity and differentiation of MDSCs. The genomic locus of miR-200c is placed in fragile regions within two chromosomal clusters of miR-200c-141 and miR-200b-200a-429 (108). PTEN, a protein/lipid phosphatase, negatively regulates the PI3K/Akt signaling pathway (109). Friend of Gata 2 (FOG2) is seen to attach to a PI3K regulatory subunit and negatively controls the PI3K/Akt pathway (110). Mei et al. demonstrated that miR-200c enhances the MDSC-mediated suppressive activity by regulating PTEN and FOG2, resulting in activation of STAT3 and PI3K/Akt. They investigated the impact of miR-200c mimics loaded MDSCs on T cell function. CD4^+^ T or CD8^+^ T cells were isolated from the spleen, labeled by CFSE and triggered with anti-CD3ε antibody, and then co-cultured with differently modified MDSCs. MDSCs treated with miR-200c mimics indicated significant inhibition on T cell proliferation with a reduction in the proportion of proliferating cells; while inhibitors of miR-200c lessened the MDSC-mediated suppressive activity. These Authors also revealed that GM-CSF has an important role in the stimulation of miR-200c in the microenvironment of tumor. Furthermore, miR-200c stimulated by GM-CSF in the tumor milieu suggests a major function in controlling the expansion and activity of tumor-related MDSCs and can be a possible target for immunomodulation in the immunotherapy of cancer (85).

### MicroRNA-9

MicrRNA-9 has been described as an important factor in controlling immune reactions (111), neuronal differentiation (112), posttraumatic stress (113), and different cancers (114, 115). Recently, Tian et al. (86) found the significant regulatory role of miR-9 in differentiation and function of MDSC by targeting Runt-related transcription factor 1 (Runx1). Runx1, as a key target of miR-9, regulates MDSC differentiation and function and is an important transcription factor during the development of hematopoietic stem cells from the hemogenic endothelium (116, 117), which is identified as a main inducer of differentiation. Runx1 controls hematopoietic stem cell differentiation into endothelial cells. It regulates the expression of some myeloid differentiation related genes and supports the differentiation of myeloid, lymphoid, and megakaryocytic lineages (116). The significant role of Runx1 in controlling MDSCs differentiation and function was confirmed by knocking down Runx1. It was found that MDSC differentiation decreased after knocking down Runx1. Moreover, suppressive agents expressed or released through MDSCs, such as arginase, iNOS, and ROS, were highly restored in MDSCs, and the ability of MDSCs to inhibit CD4^+^ and CD8^+^ T cells improved after targeting Runx1 (86, 118). These results indicated that miR-9 targeting results in decreased suppressive activity of MDSCs and increases antitumor response, which can thus be considered, with reason, a potential treatment strategy.

### MicroRNA-210

MicrRNA-210 is another important miRNA that can boost the tumor-promoting effects of MDSCs. Noman et al. (87) verified that hypoxia via HIF1a stimulates miR-210 in splenic MDSCs and increases the activity of MDSC by promoting Arg-1 expression and regulating IL-16 and CXCL12. Upregulation of miR-210 was sufficient to augment suppression of T cells by MDSC, and targeting miR-210 was enough to reduce MDSC activity against T cells. Thus, miR-210 inhibitor oligonucleotide, as an adjuvant tool, a plus novel developing immunotherapeutic approach might be helpful for improving the immune response in patients with cancer (87).

### MicroRNA-223

**MicroRNA**-223 has a central role in myeloid differentiation and is highly expressed in granulocyte lineage in human hematopoiesis (105). Its expression increasingly rises as granulocytes mature (119). Given its central role in granulocytic differentiation, the augmented expression of miR-223 is apparently able to restore differentiation in leukemic blast cells (89). In the case of MDSCs, the findings proposed a negative function for miR-223 in MDSC development because an overexpression of miR-223 can definitely control the differentiation of myeloid progenitors (90). In a murine model of autoimmune encephalomyelitis, genetic ablation of miR-223 was used to increase understanding of the molecular pathway participated in the pathogenesis of EAE. It was found that miR-223 knockout mice presented a less severe disease characterized by the enhanced accumulation of MDSC and lower levels of CNS-isolated T cell responses compared with control mice (91). A greater suppressive function detected in miR-223^−/−^ MO-MDSCs was associated with an elevated level of Arg1 and Stat3, which are reported as the target genes of miR-223 (91). The downregulation of miR-223 expression by tumor-related factors might enhance the induction and accumulation of MDSCs in the tumor milieu (90). Tumor-associated MDSCs express lower levels of miR-223 than Gr1^+^CD11b^+^ cells from the spleens of tumor-free mice. In this process, myeloid ELF1-like factor 2C (MEF2C) was detected as a direct target of miR-223, whose expression induced myeloid progenitor proliferation. MiR-223 activity suppresses the MEF2C gene that inhibits progenitor expansion, leading to decreased accumulation of MDSCs and induce tumor suppression (90).

## Long Non-coding RNAs

Long non-coding RNAs (lncRNAs) are a large class of non-coding RNAs (ncRNAs) with more than 200 nucleotides in length (120, 121). Initially characterized as “transcriptional noise” in previous RNA sequence researches, lncRNAs are currently considered as efficient RNA factors with properties of shorter length, comprising fewer exons, and low expression levels compared to messenger RNAs (mRNAs) (122–124). lncRNAs are indicated to be overexpressed in immune cells such as monocytes, macrophages, dendritic cells, neutrophils, T cells, B cells and MDSCs during their development, differentiation and activation (125). However, most of researches about lncRNAs controlling cell biology emphasize in their effects on numerous cancer cells, while their significance in MDSC regulation is also worthy of attention (Table 2).

**Table 2:** Summary of lncRNA implicated in regulation of MDSC development and function.

**LncRNA**	**Disease**	**Function**	**References**
Hox antisense intergenic RNA (HOTAIR)	Hepatocellular carcinoma	Overexpression of HOTAIR recruits TAMs/MDSCs through the release of CCL2	(126, 127)
Hox antisense intergenic RNA (HOTAIR)	Head and neck squamous cell carcinoma	HOTAIR functions as a factor in HPV16 increasing MDSC recruitment in head and neck squamous cell carcinoma	(128)
Retinal non-coding RNA3(RNCR3)	B16 tumor-bearing mice	RNCR3 stimulates differentiation and activity of MDSC through miR-185-5p/Chop	(129)
Plasmacytoma variant translocation 1 (Pvt1)	C57BL/6 tumor-bearing mice	Pvt1 augments the expression of Arg1 and ROS in G-MDSCs and decreases T-cell mediated antitumor reactions	(130)
Metastasis-associated lung adenocarcinoma transcript 1 (MALAT1)	Lung cancer	MALAT1 negatively controls MDSCs and functions as a regulator of MDSCs differentiation	(131)
RUNX1 overlapping RNA (RUNXOR)	Lung cancer	RUNXOR moderates MDSCs immunosuppression by regulating RUNX1	(132)
CCAAT/enhancer binding protein β (C/EBPβ)	C57BL/6 tumor-bearing mice	Lnc-C/EBPβ controls immune suppressive function and differentiation of MDSCs	(132)
CCAAT/enhancer binding protein β (C/EBPβ)	C57BL/6 tumor-bearing mice	Lnc-C/EBPβ moderates differentiation of MDSCs through downregulation of IL4i1	(133)
AK036396	Lung cancer	AK036396 stops maturation and accelerates immunosuppression of PMN-MDSCs by increasing the ficolin B stability	(134)

*HOTAIR, HOX transcript antisense RNA; TAM, Tumor-associated macrophages; RNCR3, Retinal non-coding RNA 3; Pvt1, Plasmacytoma variant translocation 1; MALAT1, Metastasis-associated lung adenocarcinoma transcript 1; RUNXOR, RUNX1 overlapping RNA; C/EBPβ, CCAAT/enhancer binding protein β*.

### Hox Antisense Intergenic RNA

Hox antisense intergenic RNA (HOTAIR) is a lncRNA and resembles an oncogenic molecule in several cancers. Accumulating evidence proves that HOTAIR has crucial functions in the progression and metastasis of different cancers, such as breast (135), colorectal (136), non-small lung cell (137), and gastric cancer (138). In the case of HCC cell lines, a recent study confirmed that HOTAIR-overexpressing cells release a high level of CCL2 and support macrophage/MDSC proliferation. A report demonstrated that CCL2 is an essential factor for the recruitment of macrophages and the accumulation of MDSC in the tumor milieu (81, 126, 139, 140), signifying that the overexpression of HOTAIR in cancer cells results in TAMs/MDSCs recruitment through CCL2 release, leading to augmented tumor progression and metastasis (127).

In another study, Ma et al. explained the difference in the recruitment of MDSCs seen in human papilloma virus (HPV)-positive head, neck squamous cell carcinoma (HNSCC) and HPV-negative HNSCC by using lncRNA analysis. The results showed significant function and importance of lncRNAs in HPV-associated HNSCC, demonstrating that lncRNA is a possible regulator of MDSCs recruitment in HPV-positive HNSCC (128). The level of MDSCs in HPV-positive HNSCC was notably more than in normal controls, indicating that infection with HPV can support aggregation of MDSCs. Using an array-based method to display the lncRNA expression between HPV-positive HNSCC, HPV-negative HNSCC, and normal oral mucous, 132 various lncRNAs were found in diverse HPV infected conditions of HNSCC. HOTAIR, PROM1, CCAT1, and MUC19 mRNA levels, as detected by qRTPCR, which were reversely related to MDSCs of HPV-related HNSCC. Therefore, lncRNAs resembled a modulatory factor in HPV16 supporting MDSC recruitment in HNSCC (128).

### Retinal Non-coding RNA3

Retinal non-coding RNA3 (RNCR3), also named LINC00599 is an intergenic lncRNA and is introduced as a conserved lncRNA in mammals. The proliferation and activity of various cell types can be regulated by RNCR3 (141–143). In a current research, Shang et al. proved the expression of RNCR3 in MDSCs, and it was found that this expression is considerably increased in the inflammatory and tumor microenvironment. Moreover, RNCR3 stimulates the differentiation and activity of MDSC through sponging miR-185-5p so as to produce its target gene named Chop (129). Chop is a sensor of cellular stress, which is stimulated via tumor-related ROS and nitrogen species. Chop acts as a main factor in controlling the accumulation and immunosuppressive activity of MDSCs (144). It is demonstrated that miR-185-5p affected the MDSCs expansion and reversed the impact of RNCR3 on MDSC differentiation and function by targeting Chop. Therefore, this study proposed a RNCR3/miR-185-5p/Chop autologously strengthening complex to support MDSC differentiation and suppressive activity in response to extracellular inflammatory and tumor-related signals (129).

### Plasmacytoma Variant Translocation 1

The lncRNA plasmacytoma variant translocation 1 (Pvt1) is intergenic and described as a conserved lncRNA in humans and mice. Upregulation of Pvt1 is detected in numerous cancers, such as melanoma, cervical, gastric, prostate, hepatocellular, esophageal cancers, and AML (145–147). Although lncRNA Pvt1 mechanism in cancer cells is clear, how lncRNA Pvt1 controls function and differentiation of MDSC is not well-known. A recent study indicated that Pvt1 knockdown remarkably stopped the immunosuppressive activity of G-MDSCs *in vitro*. It is shown that the Pvt1 expression is increased by HIF-1α in G-MDSCs under hypoxia. Pvt1 suppression lessened the Arg1 and ROS in G-MDSCs and increased T-cell-mediated antitumor reactions. The findings of this research demonstrated that Pvt1 targeting may reduce immunosuppression of G-MDSCs, which can be further investigated as a possible therapeutic approach (130).

### Metastasis-Associated Lung Adenocarcinoma Transcript 1

Metastasis-associated lung adenocarcinoma transcript 1 (MALAT1), a well-recognized lncRNA related to several diseases, including cancer, has increasingly received consideration In 2003, MALAT1 was firstly recognized to be significantly related to the metastasis of early-stage non-small cell lung cancer (NSCLC), and thus MALAT1 was suggested to be a predictive marker for stage I NSCLC (148). MALAT1 is a central lncRNA associated with many biological processes. The vital functions of MALAT1 in gene regulation and the significant impact on the basic activity of cells, including tumor cells, have been established by several researches (149). However, the function of this lncRNA in MDSCs is undefined. A recent research noticed the proportion of MDSCs in patients with lung cancer, the amount of ARG-1 in MDSCs, and the CD8^+^CTL cells percentage, and they also examined the MALAT1 association with MDSCs in PBMCs and revealed the regulatory activity of MALAT1 on MDSCs stimulation *in vitro*. Their results indicated a negative link between the MALAT1 level and the MDSCs proportion in PBMCs of lung cancer patients. It is suggested that MALAT1 can have a role in controlling the differentiation of MDSCs. After using MALAT1 siRNA in PBMCs, the proportion of MDSCs was meaningfully augmented as a consequence. These findings indicate that inhibition of MALAT1 promotes the quantity of MDSCs by controlling their differentiation (131).

### RUNX1 Overlapping RNA (RUNXOR)

RUNX1 overlapping RNA (RUNXOR) is introduced as an intragenic lncRNA and is about 216 kb in length. RUNXOR regulates the RUNX1 gene, which is introduced as a tumor suppressor and main regulator of hematopoiesis genes. Meanwhile, the effect of lncRNA RUNXOR on the MDSC development is uncertain (150). Tian et al. revealed the overexpression of RUNXOR and downregulation of RUNX1 in patients with lung cancer. Also, they showed that the expression of RUNXOR increases in MDSCs. After knockdown of RUNXOR, the Arg1 expression in MDSCs was downregulated. Moreover, it is indicated that after using siRUNXOR, the expression of RUNX1 is reestablished in MDSCs, and RUNX1 is negatively associated with the MDSCs proportion from patients with lung cancer. These findings presented that RUNXOR can influence the activity of MDSCs by regulation of RUNX1 (132).

### CCAAT/Enhancer Binding Protein β

The family of CCAAT/enhancer binding proteins (C/EBPs) transcription factors has vital activities in the expansion and differentiation of several types of cells such as granulocytes. C/EBPβ is informed to be essential for the maturation of monocyte and eosinophil, and currently, for neutrophil differentiation. The C/EBPβ was overexpressed according to the development of granulocytic differentiation (151, 152). LncRNA called lnc-C/EBPβ was described by Gao et al. (132) They confirmed that lnc-C/EBPβ controls numerous transcripts in MDSCs to regulate MDSC differentiation and suppressive activity in inflammatory and tumor milieus. Expression of lnc-C/EBPβ via MDSCs, negatively controls MDSC function, signifying that lnc-C/EBPβ has a negative feedback function in avoiding over-suppression of MDSCs on immune reactions (132). Moreover, it is revealed that lnc-C/EBPβ can increase polymorphonuclear MDSCs (PMN-MDSC) but inhibit the differentiation of monocytic MDSCs (Mo-MDSC). lnc-C/EBPβ can downregulate interleukin 4-induced gene-1 (IL4i1) to affect the MDSC differentiation by attaching with C/EBPβ LIP and WD repeat-containing protein 5 (WDR5) (133).

### AK036396

As the largest group of MDSCs, PMN-MDSCs indicate a fundamental role in increasing the immune escape of numerous cancers. Tian et al. recognized that lncRNA AK036396 and its target Ficolin B (Fcnb) were most abundant in PMN-MDSCs, compared with other myeloid cells. They detected the regulatory mechanism of lncRNA AK036396 in the PMN-MDSCs. Downregulation of LncRNA AK036396 decreased Fcnb protein stability in an ubiquitin-proteasome system-dependent mechanism. They revealed that the downregulation of lncRNA AK036396 stimulated the maturation and reduced the suppressive activity of PMN-MDSCs. Moreover, the expression of human M-ficolin, which is an ortholog of mouse Fcnb, was augmented and associated with arginase1 expression (134).

## Concluding Remarks

MDSCs are immature immunosuppressive cells that are involved in a wide spectrum of diseases and in particular in cancer. The development of MDSC-targeted approaches to improve immunotherapy appears to be a high priority and might bring new results in the field of immunotherapy. One considerable challenge is the complexity of factors that are found to play a critical role in regulating MDSC function and expansion. Increasing evidence suggests that miRNAs and lncRNAs are implicated in the pathogenesis of diseases or are at least linked to the disease state. Moreover, the aberrantly expressed miRNAs and lncRNAs were significantly associated with MDSC development and function. In addition to intracellular regulation, miRNAs and lncRNAs can show intercellular impacts via exosomes (153). Exosomes are released by diverse cell types, including cells with a myeloid lineage, such as MDSCs. Inadequate evidence on MDSC exosomes reveals that they can exert effects related to immunosuppression and the elevation of tumorigenesis (154). It is presented that exosomes were secreted more plentifully from tumor MDSCs, apparently due to the fact that MDSCs in the TME are in a more stressful environment (155). Furthermore, tumor exosomes have been informed to carry genetic materials and proteins capable of suppressing the immune cells functions and stimulating the activation and expansion of MDSCs *in vitro* and *in vivo* (156). It is shown that upregulation of miR-10a and miR-21 in hypoxia-induced glioma-derived exosomes has a powerful impact in MDSC induction. Hypoxia-inducible miR-10a and miR-21 expression in glioma-derived exosomes increased the expansion and suppressive activities of MDSCs. These findings demonstrate that exosomes have a prognostic value, promising positive inputs for new therapeutic directions in the cancer treatment. The promise of targeting miRNAs and lncRNAs, by either antagonizing or restoring function, might provide novel strategies to re-educate immunosuppressive or hyperactive milieu of disease associated with MDSC development, including cancer. Given the heterogeneity of human MDSCs and limitation in clinical application, it is reasonable to predict that more information is urgently needed, and new ideas and questions are proposed for future inquiry.

## Author Contributions

ES and ZA: conception, design, and writing. SS, AH, AD, FG, and OB: literature search and writing. NS and BB: editing and correction. All authors contributed to the article and approved the submitted version.

## Conflict of Interest

The authors declare that the research was conducted in the absence of any commercial or financial relationships that could be construed as a potential conflict of interest.
